# Innovative Solid Slippery Coating: Uniting Mechanical Durability, Optical Transparency, Anti-Icing, and Anti-Graffiti Traits

**DOI:** 10.3390/polym15193983

**Published:** 2023-10-03

**Authors:** Jiayi Shen, Junfei Ou, Sheng Lei, Yating Hu, Fajun Wang, Xinzuo Fang, Changquan Li, Wen Li, Alidad Amirfazli

**Affiliations:** 1 School of Materials Engineering, Jiangsu University of Technology, Changzhou 213001, China; 15821116785@163.com (J.S.); shenglei@jsut.edu.cn (S.L.); huyt@jsut.edu.cn (Y.H.); wangfj@jsut.edu.cn (F.W.); 2017500077@jsut.edu.cn (X.F.); 2017500057@jsut.edu.cn (C.L.); 2017500100@jsut.edu.cn (W.L.); 2 Department of Mechanical Engineering, York University, Toronto, ON M3J 1P3, Canada; alidad2@yorku.ca

**Keywords:** solid slippery coating, durability, anti-freezing, anti-graffiti, anti-smudge

## Abstract

Slippery coatings, such as the slippery liquid-infused porous surface (SLIPS), have gained significant attention for their potential applications in anti-icing and anti-fouling. However, they lack durability when subjected to mechanical impact. In this study, we have developed a robust slippery coating by blending polyurethane acrylate (PUA) with methyltriethoxysilane (MTES) and perfluoropolyether (PFPE) in the solvent of butyl acetate. The resulting mixture is homogeneous and allows for uniform coating on various substrates using a drop coating process followed by drying at 160 °C for 3 h. The cured coating exhibits excellent water repellency (contact angle of ~108° and sliding angle of ~8°), high transparency (average visible transmittance of ~90%), exceptional adherence to the substrate (5B rating according to ASTMD 3359), and remarkable hardness (4H on the pencil hardness scale). Moreover, the coating is quite flexible and can be folded without affecting its wettability. The robustness of the coating is evident in its ability to maintain a sliding angle below 25° even when subjected to abrasion, water jetting, high temperature, and UV irradiation. Due to its excellent nonwetting properties, the coating can be employed in anti-icing, anti-graffiti, and anti-sticking applications. It effectively reduces ice adhesion on aluminum substrates from approximately 217 kPa to 12 kPa. Even after 20 cycles of icing and de-icing, there is only a slight increase in ice adhesion, stabilizing at 40 kPa. The coating can resist graffiti for up to 400 cycles of writing with an oily marker pen and erasing with a tissue. Additionally, the coating allows for easy removal of 3M tape thereon without leaving any residue.

## 1. Introduction

Bioinspired surfaces, such as a superhydrophobic surface [1] and slippery liquid-infused porous surface (SLIPS) [2,3,4], exhibit similarities in their water-repellent properties. Aizenberg et al. drew inspiration from nepenthe and developed the SLIPS by introducing a low-surface-energy lubricating liquid into porous surface structures. This process resulted in the formation of a continuous and smooth liquid lubricant film, enabling water droplets to effortlessly slide off, even at a small contact angle [5,6,7]. Due to the excellent water repellency and low ice adhesion strength, the SLIPS demonstrated great potential in anti-icing applications [8,9,10,11]. For instance, Wilson et al. [12] found that the SLIPS could significantly reduce the nucleation temperature of supercooled water in contact, with statistical significance, and showed no deterioration or change in the coating performance even after 150 freeze–thaw cycles. Liu et al. [13] proposed a novel self-assembly method of fabricating an electric heating SLIPS, which possessed ultra-low ice adhesion. Mahmut et al. [14] fabricated three kinds of SLIPS with the lubricants polychlorotrifluoroethylene oil, silicone oil, and liquid paraffin. All the SLIPS revealed extremely low ice adhesion of lower than 1 kPa.

The fabrication of the SLIPS typically involves three primary steps: (1) creating rough surface structures; (2) applying a layer of low-surface-energy molecules to passivate the rough surface; and (3) impregnating the surface with liquid lubricants [2,15,16]. The rough surface structures generated in the first step serve as a physical reservoir for the liquid lubricant, while the second step employs low-surface-energy molecules to chemically attract the lubricant. For instance, Sun et al. [17] used electrochemical etching and anodization to create a hierarchical porous structure on the substrate Al. After lowering the surface energy and infusing silicone oil, the SLIPS was obtained, showing a water roll-off angle of 3°. Yan et al. [18] synthesized titanium dioxide nanotube arrays on titanium substrate through the anodic oxidation method, which were further modified and infused with perfluoropolyether lubricant to form the SLIPS. The contact angle hysteresis and sliding angles of water droplets on the TiO_2_ SLIPS were as low as 1° and 4°, respectively, indicating the excellent slippery property.

The inherent weakness of the SLIPS is that the lubricating oil can be lost easily, leading to a decline in sliding performance. To enhance the durability of the SLIPS, Tan et al. [19] fabricated a fractal surface with micro-sized pyramid holes and porous nanostructures. The porous nanostructures served to retain the lubricant perfluoropolyether whilst the robust micro-pyramidal holes provided protection for the nanostructures. The other solution to enhance the durability of SLIPS is to bond the lubricant to the substrate via affinity bonding. For example, Wu et al. [20] developed a liquid-attached SLIPS via a one-step equilibration reaction by tethering methoxy-terminated polydimethylsiloxane polymer brushes onto a substrate to form a transparent “liquid-like” layer. The so-obtained surface demonstrated superior abrasion resistance and maintained its excellent self-cleaning capacity even after being subjected to abrasion with water or sand particles. Solid lubricant such as paraffin wax was also used for the robust slippery coating. For instance, Meng et al. [21] developed a coating by incorporating paraffin wax into a porous polystyrene structure. This solid slippery surface offered remarkable stability, even when exposed to various pH solutions or contact with other materials. Additionally, it demonstrated rapid self-healing capabilities under the heating–cooling process. However, the fabrication procedure was complex and not conducive to large-scale production. Moreover, the use of porous polymers restricted the choice of substrate materials to polymers, thereby limiting the application range.

This study introduces a novel robust slippery coating that is synthesized using a one-pot method. To form the coating film, polyurethane acrylate is chosen as the film-forming material. In order to enhance water repellency and stability, a combination of methyltriethoxysilane (MTES) and perfluoropolyether (PFPE) is incorporated. The resulting coating exhibits exceptional durability, making it suitable for long-term use. Its low surface energy also allows it to effectively resist ice formation and repel stains.

## 2. Experimental Procedure

### 2.1. Materials

Polyurethane acrylate (PUA, WDS-9800, 98 wt.%) was obtained from Wuxi Weidusi Electronic Materials Co., Ltd. (Jiangsu, China). Perfluoropolyether (Krytox, GPL 105, 99.9%) was purchased from DuPont. Methyltriethoxysilane (MTES) and the solvent butyl acetate were all of analytic grade and purchased from Aladdin Chemical Co., Ltd. (Shanghai, China).

### 2.2. Coating Fabrication

To create a uniform and well-mixed solution, the following components were combined: 10 g of PUA, 1.5~6 g of MTES (with the optimum amount being 4.5 g), and 0.04~0.1 g of PFPE (with the optimum amount being 0.18 g). These ingredients were added to 10 g of butyl acetate. The mixture was stirred for 30 min to ensure thorough mixing. Following the preparation of the precursor solution, 1 mL of it was carefully poured onto a clean glass slide sized 25.4 mm × 76.2 mm, and the solution spread spontaneously across the surface of the substrate. To ensure proper solidification, the coated slide was dried at a temperature of 160 °C for a duration of 3 h. The drying process helped to solidify the coating and ensure its durability.

### 2.3. Characterization

The wettability was evaluated using a contact angle meter (DSA30, Krüss, Hamburg, Germany) with ultrapure water as the probe liquid. Fourier transform infrared spectroscopy (Nicolet IS5, Thermo Fisher Scientifics, Woltham, MA, USA) was used to study the chemical composition of the coating. Light transmittance was measured using an ultraviolet-visible spectrophotometer (G6860A, Agilent Technologies, Santa Clara, CA, USA). Surface morphology was observed using atomic force microscopy (5500, Agilent, USA) and field emission scanning electron microscopy (FE-SEM, Novananosem, FEI, Hillsboro, OR, USA). The adhesion test was implemented on a coated glass plate according to the ASTM D3359 standard. The hardness of the coating was determined using a pencil hardness test according to the ASTM D 3363-74 standard.

### 2.4. Durability Tests

The durability of the coating was assessed by examining its surface wettability after tests. The abrasion test was conducted using an abrasion tester (ZJ-339-GSR, Yingshang Zhuoyue, China) against medical gauze under a load of either 1.25 kPa or 5 kPa. One cycle constitutes back and forth abrasion for a distance of 10 cm at a speed of approximately 6 cm/s. The heat resistance test was performed in a furnace (KSL-1100X, Hefei Kejing, Hefei, China) up to 400 °C for 1 h. The UV resistance was evaluated by exposing the coating to a UVB ultraviolet lamp (with the maximum radiation wavelength of 313 nm and a power of 30 W) at a distance of 5 cm for 7 days. To evaluate the chemical resistance of the sample to acid or alkali, a simulated rain test was performed. The sample was positioned at a 45° angle and exposed to simulated acid rain with a pH of 5 (adjusted using HCl) or alkali rain with a pH of 9 (adjusted using NaOH). For the test, a plastic bottle with small holes at the bottom was used to dispense the acid or alkali solution onto the sample. The bottle was positioned above the sample at a height of 35 cm. The holes in the bottle were spaced approximately 3 mm apart, and each hole had a diameter of around 1 mm. During each cycle of the test, 100 mL of the acid or alkali solution was dispensed onto the sample, simulating rainwater exposure. This allowed for the assessment of the sample’s resistance to acid or alkali under controlled conditions. In the water jetting test, the sample was positioned at a 30° angle relative to the horizontal plane. A water flow was directed towards the sample with a rate of 1.1 L/min and a velocity of 2 m/s. The distance between the water source and the sample was maintained at 30 cm.

### 2.5. Ice Adhesion Test

To assess the adhesion strength of the coating, a lyophilizer set to a low temperature of −30 °C was utilized. A container with dimensions of 1 cm × 1 cm × 1 cm was placed on the coating and filled with deionized water until it completely froze. The frozen sample was then carefully removed from the lyophilizer and transferred onto a Peltier cooling stage to maintain a low surface temperature. To test the adhesion strength, a tensimeter was employed as quickly as possible. The sample was subjected to a tensile force until the ice coating detached from the surface. To ensure accurate measurement results, the ice adhesion strength was tested three times, and the average value was calculated to obtain a more reliable assessment of the coating’s adhesion properties.

### 2.6. Anti-Graffiti and Anti-Sticking Test

To evaluate the anti-graffiti property of the coating, it was marked with an oily marker (Deli, China). The marks were then wiped off with tissue paper after drying for one minute. This process was repeated multiple times to assess the coating’s durability and resistance to graffiti. To assess the anti-sticking ability of the coating, pressure was applied with the fingers to adhere 3M 55236 adhesive tape onto it, followed by removal to evaluate the ease of peeling.

## 3. Results and Discussion

### 3.1. Component Optimization and Surface Characterization

**Figure 1:**
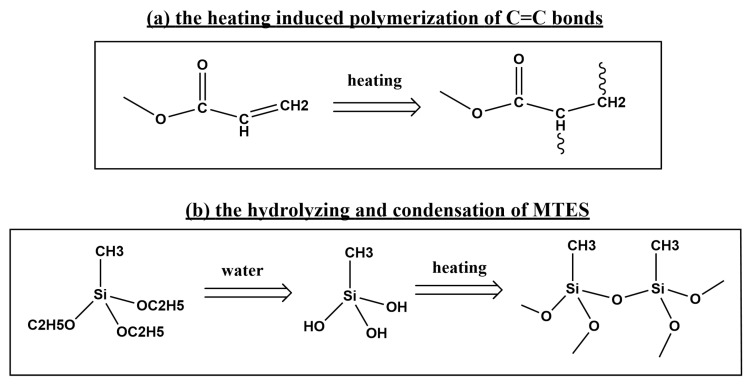
Curing mechanism of the solid slippery coating.

The C=C bonds within the acrylate moiety dissociated under heat, leading to the formation of a polymer structure (as shown in Figure 1a). Simultaneously, heat-induced dehydration condensation took place among the hydrolyzed MTES molecules, resulting in the formation of a closely linked Si-O-Si network (as shown in Figure 1b) [22]. This network significantly enhances the crosslinking density of the coating and improves its durability and overall performance, which will be elaborated upon in the upcoming sections. The incorporation of PFPE into the coating formulation allowed it to intertwine among the polymer chains, resulting in increased slipperiness to water droplets. This combination of robustness and slipperiness contributed to the achievement of a coating that was both durable and offered improved slip behavior.

**Figure 2:**
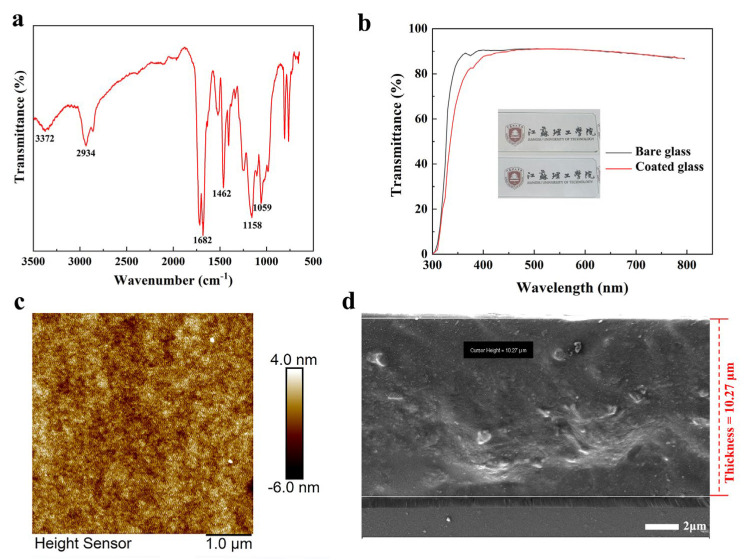
(**a**) Fourier transform infrared spectrum of the slippery coating. (**b**) Optical transmittance spectra of the slippery coating on glass measured in the wavelength range from 300 nm to 800 nm. Inset images show a comparison between the bare glass and the glass coated with the slippery coating. (**c**) Surface morphology of the slippery coating on glass observed using an atomic force microscope. (**d**) Thickness of the slippery coating observed through cross-section analysis using a scanning electron microscope.

The results obtained from the infrared spectroscopy (FTIR) analysis (Figure 2a) provide valuable insights into the chemical composition of the coatings. The stretching vibration peaks located at 3372 cm^−1^ (N-H) and 1682 cm^−1^ (C=O) indicate the presence of urethane functional groups. The absorption peaks at 2934 cm^−1^ and 1462 cm^−1^ were attributed to the stretching and bending vibrational features of C-H and -CH_3_ groups, respectively. Additionally, the stretching vibrational absorption peak observed at 1059 cm^−1^ indicated the successful incorporation of MTES, as it was assigned to Si-O-Si bonds. Furthermore, the asymmetric stretching vibrational absorption peak at 1158 cm^−1^ was linked to C-F bonds, confirming the successful introduction of PFPE into the coating formulation. Overall, the FTIR analysis provides evidence of the presence of specific functional groups and the successful incorporation of key components within the coating.

The transmittance of the coating on the glass was measured using a UV-VIS spectrophotometer, revealing an average transmittance of 89.7% within the wavelength range of 400–800 nm. This value was quite similar to that of the bare glass (90%, Figure 2b). The coating’s transparency allowed for clear visibility of the school emblem of Jiangsu University of Technology, which was placed under the coating on the glass substrate (inset in Figure 2b). An atomic force microscope was used to measure the surface morphology of the coating. The coating exhibited a smooth and compact surface with minimal height differences between peaks, as demonstrated by a surface roughness of only 1.02 nm (Figure 2c). Furthermore, SEM analysis revealed a thickness of approximately 10 μm when performing cross-sectional analysis (Figure 2d).

**Figure 3:**
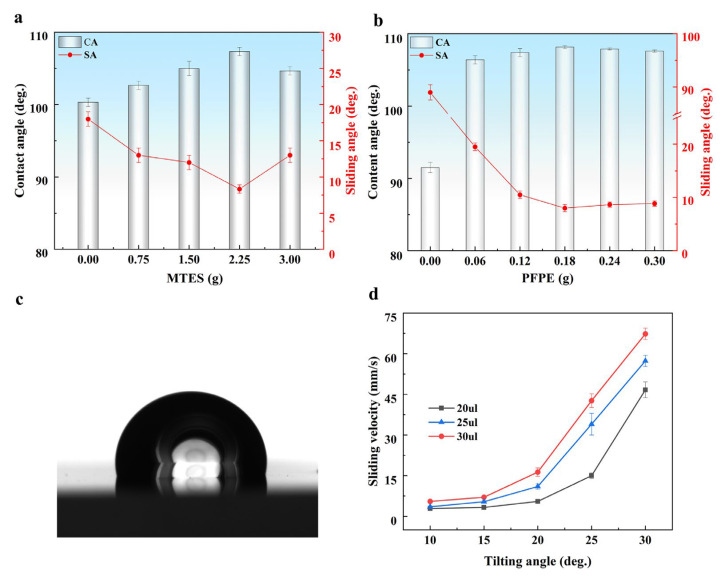
Variation in surface wettability of the slippery coating against the weight of (**a**) MTES and (**b**) PFPE in the precursor solution. (**c**) Snapshot of a water droplet on the slippery coating with the contact angle of 108°. (**d**) Variation in the sliding velocities of water droplets with different volumes on the inclined coating at different tilt angles. The results in (**c**,**d**) are for the coating with the optimized formulation of MTES (1.5 g) and PFPE (0.18 g).

The influence of MTES content on the sliding performance of the coating was investigated (Figure 3a). The weight of PFPE in the mixture was kept constant at 0.18 g. In the absence of MTES, the sample contained only PUA and PFPE, exhibiting hydrophobic properties with a contact angle of 100° and a sliding angle of approximately 17°. As the MTES content increased, there was an initial rise followed by a decline in the contact angle. Similarly, the sliding angle initially decreased and then increased. This behavior can be attributed to the presence of two types of groups in MTES: the non-polar CH_3_ group and the polar -OC_2_H_5_ group. The latter group can be hydrolyzed in the presence of water, giving rise to hydrophilic −OH groups and Si-O-Si groups (Figure 1b). Thus, in the initial stage of increasing the MTES amount, the non-polar CH_3_ group dominates, resulting in an increase in the contact angle. However, once the MTES amount exceeds 4.5 g, the hydrophilic groups become dominant, causing a decrease in the contact angle. Following the optimization of the MTES content at 4.5 g, the effect of varying the PFPE content on the water droplet sliding performance of the solid slippery coating was examined. The optimal performance was achieved when the weight of PFPE was 0.18 g, resulting in a contact angle of approximately 108° and a sliding angle of approximately 8° (Figure 3b). Subsequently, sliding speed tests were conducted on the coating, demonstrating that water droplets could easily slide off, with the sliding speed increasing as the tilt angle increased (Figure 3d).

### 3.2. Durability and Protection Performance

**Figure 4:**
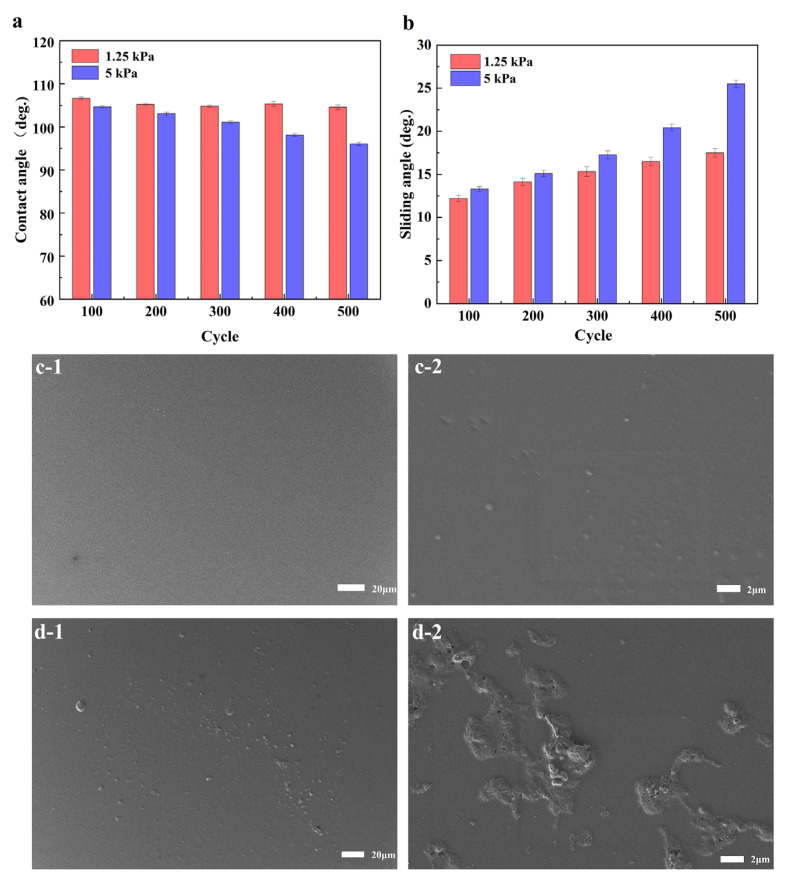
Variation in contact angle (**a**) and sliding angle (**b**) over different numbers of abrasion cycles and under different loads for the coating on the glass substrate. SEM images for the coating after abrasion for 500 cycles under a load of 1.25 kPa (**c-1**,**c-2**) or 5 kPa (**d-1**,**d-2**).

The durability of the slippery coating on the glass substrate was assessed using various tests [23]. Rubbing tests were employed to evaluate its mechanical durability. The results showed that, after 500 cycles of rubbing against medical gauze at a load of 1.25 kPa, the contact angle of the coating decreased slightly from around 108° to approximately 105° (Figure 4a) and the sliding angle increased from 8° to 17.5° (Figure 4b). It is worth noting that despite these changes, there were no visible wear scars on the coating’s surface; only scattered fine particles were observed after 500 cycles (Figure 4c-2). When subjected to a higher load of 5 kPa, the contact angle remained relatively high at around 95°, while the sliding angle increased to approximately 25°. After 500 friction cycles at this load, the coating exhibited slight wear scars along with debris (Figure 4d). Although there was a slight alteration in the microscopic morphology of the coating due to rubbing, there were no observable changes in its macroscopic appearance. In summary, the slippery coating demonstrated satisfactory mechanical durability during the rubbing tests, as there were minimal visible wear scars and particle accumulation even after a significant number of cycles.

**Figure 5:**
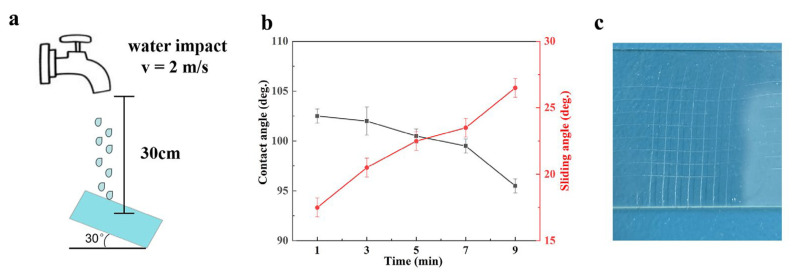
(**a**) Key parameters for the water jetting test. (**b**) Variation in the contact angle and the sliding angle against the water jetting time. (**c**) Macro surface appearance of the slippery coating on the glass after the cross-cutting and tape peeling test.

Key parameters for the water jetting test are shown in Figure 5a. After 9 min, a water droplet (20 μL) could still easily slide off the tiled sample at a 30° angle to the horizontal surface (Figure 5b). The slippery coating was highly resilient, exhibiting a pencil hardness level of 4H. To assess the adhesive strength of the slippery coating, we followed the ASTM D 3359 standard and employed a cross-cut tester to create a grid pattern on the slippery coating. Subsequently, we applied adhesive tape to the grid and peeled it off, ultimately observing no detachment of the coating from the substrate. This outcome indicated a robust adhesive strength of 5B between the coating and the substrate (Figure 5c).

**Figure 6:**
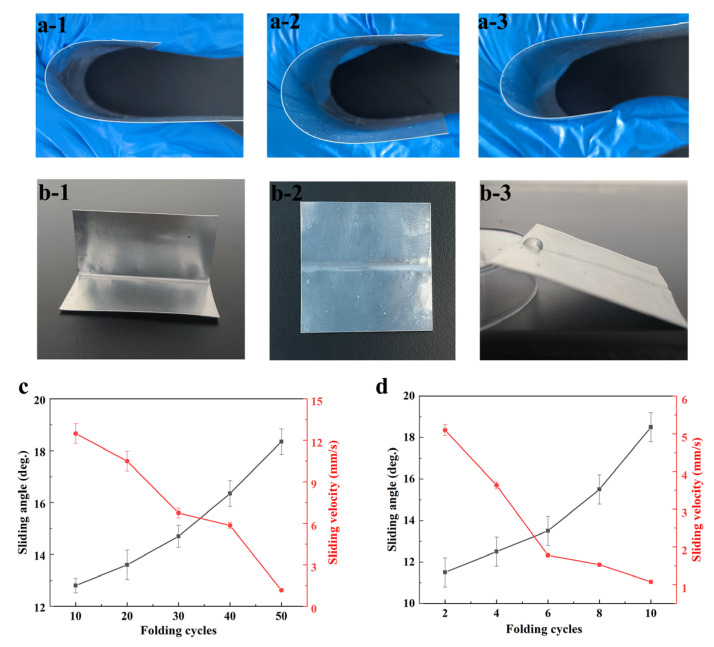
Snaps of the coated PET film being folded into a "U" shape (**a-2**) and moved back (**a-1**) and forth (**a-3**). (**b**) Snaps of the coated Al plate folded at a 90° angle (**b-1**) and then restored to its original state (**b-2**). A water droplet with the volume of 20 µL is sliding on the crease with the tilting angle of 20° (**b-3**). Variation in sliding angle and sliding velocity of the coated PET (**c**) and Al plate (**d**) with different folding times. A 20 µL water droplet was tested on the coated substrate and the tilting angle was 20°.

We performed tests to evaluate the flexural resistance of the coating on PET films and aluminum sheets. In the case of PET films, a “U” shape was formed by bending the film and moving it back and forth to assess its flexural resistance, as depicted in Figure 6a [24]. After bending, the sliding angle and sliding velocity of a 20 μL water droplet, tilted at an angle of 20°, were measured. Figure 6c illustrates that after 10 bending cycles, there was only a slight increase in the sliding angle of the coating (13°), while the sliding speed remained relatively fast (12 mm/s). After 50 bending cycles, the coating remained intact and the sliding angle increased to 19°, while the sliding speed decreased to 1.8 mm/s. The flexural resistance of the coating on aluminum was assessed by folding the aluminum sheets at a 90° angle, followed by restoration. After 10 bending–restoration cycles, no cracking was observed on the bent area of the coating. In Figure 6d, we present the results of tests examining the ability of water droplets to smoothly slide off the crease. The coating exhibited a sliding angle lower than 20° and a sliding velocity of approximately 1 mm/s, indicating its favorable flexural resistance. These findings demonstrate the durability and performance of the coating on PET films and aluminum sheets under bending conditions, making it suitable for various applications.

**Figure 7:**
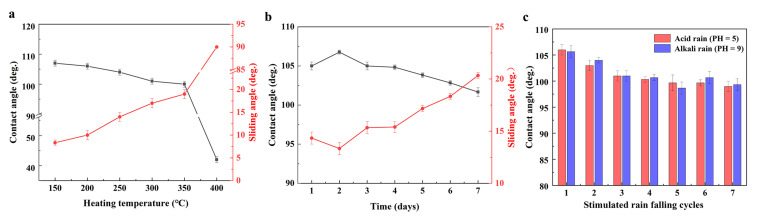
Variation in the contact angle and sliding angle against the heating temperature (**a**) (the heating duration is 1 h) and the UV radiation time (**b**) for the composite slippery coating. (**c**) Variation in contact angle against the simulated rain cycles for the composite slippery coating.

After storage in a furnace for 1 h at 350 °C, the contact angle of the slippery coating slightly decreased to approximately 102°, and the sliding angle increased to approximately 18° (Figure 7a). Upon continuous heating to 400 °C, the contact angle of the slippery coating surface sharply dropped to 42°, and the sliding angle increased to be higher than 90°. The high sliding angle indicated that the water droplet could not slide off, even when the slippery coating was vertically placed. When the slippery coating was exposed to UV irradiation (313 nm, 30 W) at a distance of 5 cm, the contact angle decreased to 103° and the sliding angle increased to approximately 20° after 7 days. To simulate the impact of acid rain and alkaline rain, the slippery coating was subjected to water droplets of an aqueous solution of HCl and NaOH [25]. After seven cycles, the contact angle remained at a high level of approximately 95°. However, the water droplets could not easily slide off the surface.

The results obtained from the experiments were consolidated into Table 1 and compared with typical reference samples [9,10,23,24,26,27,28,29,30,31,32]. The findings revealed that the slippery coating developed in this study demonstrates exceptional performance in various aspects, including optical transparency, mechanical durability, flexibility, chemical robustness, and UV resistance. These remarkable characteristics render it ideal for practical applications, such as anti-ice, anti-graffiti, and anti-sticking purposes, which will be discussed in detail shortly.

### 3.3. Applications: Anti-Icing, Anti-Graffiti and Anti-Sticking

**Figure 8:**
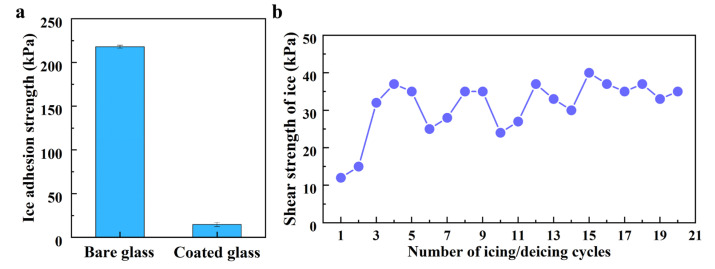
(**a**) Adhesion strength of ice on the bare glass and the slippery coating. (**b**) Variation in the adhesion strength of ice on the slippery coating against the icing/de-icing cycles.

The accumulation of ice on surfaces can cause significant problems, and current de-icing methods have limitations in effectively removing ice. To address this issue, a slippery coating was utilized to reduce ice adhesion. Without the coating, the glass surface demonstrated a high ice adhesion strength of approximately 217 kPa (Figure 8a). However, when the slippery coating was applied, the ice adhesion strength dropped significantly to 12 kPa. It is worth noting that ice adhesion below 20 kPa allows for easy removal [33]. Although similar low ice adhesion has been observed in previous studies on SLIPS [34], ensuring long-term durability for an anti-icing SLIPS remains a challenge. To evaluate the durability of the slippery coating, multiple ice formation/removal cycles were conducted. Even after 20 cycles, the ice adhesion strength of the coating remained between 30 and 40 kPa (Figure 8b). This indicates that the slippery coating exhibits exceptional mechanical strength, enabling it to withstand the expansion stress caused by freezing. Furthermore, compared to previously reported slippery coatings [8,9,11,35,36], our slippery coating demonstrates comparable anti-icing performance and superior durability (Table 2).

**Figure 9:**
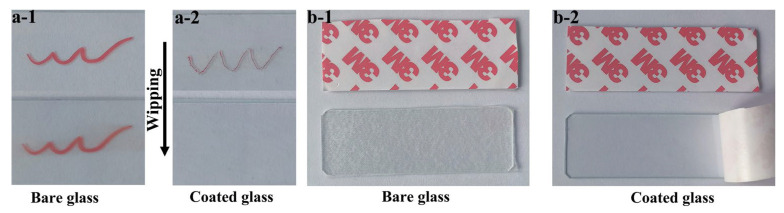
Comparison of anti-graffiti performance between bare glass (**a-1**) and the slippery coating after 400 wiping cycles (**a-2**). Evaluation of anti-sticking performance on bare glass (**b-1**) and the slippery coating (**b-2**).

The anti-smudge properties of the developed slippery coating were evaluated, particularly with regards to oily markers. It should be noted that high contact angles and low sliding angles of water on the coating surface do not necessarily indicate anti-smudge capabilities, as the contraction of oil-based ink on the coating poses a greater challenge. In the case of an insufficiently cross-linked coating surface, the ink may exhibit minimal or no contraction behavior, resulting in persistent and noticeable marks even after wiping. When an oily marker was used on glass, visible lines remained on the surface that were not easily removed by simple wiping with a dry tissue (Figure 9a-1). However, the slippery coating demonstrated excellent anti-graffiti properties. The weak interaction between the ink and the coating caused the ink to form a discontinuous line, allowing for easy erasure with a tissue [37]. Even after 400 cycles of writing and erasing (as shown in Figure 9a-2), the anti-graffiti performance remained unaffected, making it highly practical for real-world applications.

Additionally, the slippery coating proved highly effective in resisting adhesion to solid adhesives. As illustrated in Figure 9b, the 3M adhesive tape adhered firmly to the bare glass, making it difficult to peel off. However, when applied to the slippery coating, the 3M tape could be effortlessly removed without leaving any residue on the sample.

## 4. Conclusions

A multifunctional and robust slippery coating was developed using polyurethane acrylate, methyltriethoxysilane, and perfluoropolyether. The smooth surface morphology of the coating resulted in impressive slipperiness, with a contact angle of approximately 108° and a sliding angle of around 8°. Significantly, the coating maintained its effectiveness even after exposure to challenging conditions, such as high temperatures or low humidity. It also exhibited exceptional hardness (4H) and substrate adherence (5B), making it highly resistant to water-jet impacting and abrasion, showcasing its durability. The coating demonstrated excellent optical transparency and remarkable performance in terms of graffiti resistance and prevention of sticking. Additionally, its low shear strength against ice made de-icing effortless. This versatile coating holds great potential for applications in anti-icing and anti-smudge scenarios. Overall, this work represents a significant step forward in the development of a versatile and durable slippery coating with multiple benefits for various applications. Future directions for this work may include the development of such coatings through room temperature curing, which could lead to more efficient and environmentally friendly manufacturing processes.

## Figures and Tables

**Table 1:** Behavior of the coating fabricated in our work as compared to references. CA and SA are the abbreviations of contact angle and sliding angle, respectively.

Test	Testing Standard or Details and the Results	Ref
Visible light transmittance	88%	[9]
	85%	[10]
	73.1%	[26]
	89.7%	This work
Abrasion resistance	SA increased to 25° after 20 cycles of abrasion. Abrasion distance of 10 cm each cycle with a load of 10 kPa against 1000-mesh sandpaper	[27]
	SA increased to 8° after 10 cycles. Abrasion distance of 20 cm each cycle with a load of 1 kPa against 1500-mesh sandpaper	[28]
	SA increased to 12° after 11 cycles. Abrasion distance of 20 cm each cycle with a load of 100 g against 1000-mesh sandpaper	[29]
	CA decreased to 95° and SA increased to 25° after 500 cycles. Abrasion distance of 10 cm each cycle with a load of 5 kPa against medical gauze	This work
Water jetting	Sample was tilted at a 30° and jetted with water at 2 m/s for 1 min. After the test, water droplets slid off easily	[23]
	Sample was tilted at a 45° and jetted with water at 1.34 m/s for 5 min. After the test, water droplets slid off easily	[26]
	Sample was tilted at a 45° and jetted with water at 1.5 m/s for 5 min. After the test, the sample lost its slippery performance	[29]
	Sample was tilted at a 30° and jetted with water at 2 m/s for 7 min. After the test, water droplets slid off easily	This work
High temperature resistance	Almost no change in CA, while SA increased to 50° after 10 min at 240 °C	[23]
	CA decreased to 65° and SA increased to 3.5° after 15 min at 220 °C	[30]
	CA decreased to 102° and SA increased to 19° after 1 h at 350 °C	This work
Bending	No cracking observed after 20 cycle of bending the coating on aluminum	[24]
	No cracking observed after 1 cycles of bending the coating on PET film	[31]
	No cracking observed after 50 cycles of bending the coating on PET film No cracking observed after 10 cycles of bending the coating on aluminum	This work
Pencil Hardness	3 H	[23]
	3 H	[32]
	4H	This work

**Table 2:** Adhesion strength of ice on the slippery coating in this study and on the typical samples in the references.

Ref. No.	Icing and De-Icing Cycle	Adhesion Strength (kPa)	Adhesion Strength after Icing and De-Icing Cycles (kPa)
[8]	4	22	32
[9]	10	14	14
[11]	7	10	50
[35]	15	5	18
[36]	16	40	90
This work	20	12	30~40

## Data Availability

Data will be made available on request.
